# Real-World Evaluation of a Near-Infrared Spectroscopy–based Noninvasive Glucose Monitoring Device in Adults With Type 2 Diabetes

**DOI:** 10.1177/19322968261496268

**Published:** 2026-10-25

**Authors:** Sandra Amuedo, Mónica Enríquez Macías, José Manuel Fornés, Arturo Moreno Arteaga, Jesús Riesgo Barrau, Virginia Bellido, Fernando Losada Viñau, Luz María Hidalgo Pichardo, Alfonso Soto Moreno, Antonio Puppo Moreno, Alfonso Pumar López

**Affiliations:** 1 Unidad de Gestión Clínica de Endocrinología y Nutrición, Hospital Universitario Virgen del Rocío, Sevilla, Spain; 2 Instituto de Biomedicina de Sevilla (IBiS), Hospital Universitario Virgen del Rocío/CSIC/Universidad de Sevilla, Spain; 3 Telematic Engineering Department, University of Seville, Spain; 4 GLUCUBE, Seville, Spain; 5 Unidad de Gestión Clínica de Cuidados Intensivos, Hospital Universitario Virgen Del Rocío, Sevilla, Spain

**Keywords:** glucose monitoring accuracy, near-infrared spectroscopy, noninvasive glucose monitoring, self-monitoring of blood glucose, type 2 diabetes, usability

## Abstract

**Background:**

Self-monitoring of blood glucose remains widely used in many individuals with type 2 diabetes (T2D), although the burden of capillary testing often limits the frequency of measurements. Noninvasive technologies may improve glucose monitoring. This study evaluated the analytical performance and applicability of a near-infrared spectroscopy–based noninvasive glucose monitoring device.

**Methods:**

In this prospective, open-label clinical investigation, 52 adults with T2D performed 4 daily paired glucose measurements for more than 21 days using the investigational device and a reference capillary glucometer (Ascensia Contour Next). Following individualized calibration (10 paired measurements), post-calibration measurements within the 70 to 400 mg/dL range were included in the primary analysis. Accuracy was assessed using a combined mean absolute difference/mean absolute relative difference (MAD/MARD) metric, Parkes consensus error grid (CEG), and DTS error grid analyses.

**Results:**

Forty-eight participants were included in the primary analysis, contributing 2,616 paired post-calibration measurements. The mean age was 58.0 ± 9.9 years, and 31.3% were female. The overall combined MAD/MARD value was 16.3, calculated by applying MAD to paired measurements with reference glucose values <100 mg/dL and MARD to paired measurements with reference glucose values ≥100 mg/dL. Overall, 69.8%, 86.3%, and 94.5% of measurements were within ±20%, ±30%, and ±40% of reference values, respectively. Parkes CEG analysis classified 69.9% of measurements in zone A, 29.1% in zone B, 1.0% in zone C, and none in zones D or E. Accuracy improved after the first week. No device-related adverse events were reported.

**Conclusion:**

The device demonstrated a favorable clinical risk profile, stable analytical performance, and good safety under real-world conditions. Although analytical precision remains lower than current continuous glucose monitoring systems, its fully noninvasive design supports further development as a complementary monitoring strategy for individuals with T2D unwilling or unable to use invasive monitoring technologies.

**Trial Registration:**

ClinicalTrials.gov (NCT07075796).

## Introduction

Despite the established benefits of glucose monitoring, adherence to self-monitoring of blood glucose (SMBG) remains suboptimal in many individuals with T2D because of the burden associated with capillary fingerstick testing, including discomfort, inconvenience, and cost.^1-3^ Consequently, a substantial proportion of individuals with T2D remain under-monitored in routine clinical practice.

Continuous glucose monitoring (CGM) systems have improved glucose assessment and demonstrated important clinical benefits.^4,5^ However, barriers such as sensor insertion, skin-related adverse effects, device burden, and cost may still limit their use in some individuals with T2D. As a result, additional monitoring approaches that are accessible and easy to use remain desirable.

Near-infrared spectroscopy (NIRS) has emerged as a promising approach for noninvasive glucose monitoring by estimating glucose concentrations through optical tissue measurements. Although clinical implementation has historically been limited by technical challenges related to signal quality and biological variability, advances in signal processing and calibration algorithms have renewed interest in this technology.^6^

GLUCUBE (IGLUCO TECH, S.L., Seville, Spain) is an NIRS-based noninvasive glucose monitoring device that combines spectroscopic measurements with individualized calibration algorithms to generate glucose estimates. The individualized calibration required 10 paired measurements performed during the initial 2 to 3 days of device use to establish participant-specific calibration parameters. Following this calibration phase, the device provides on-demand glucose estimations without routine fingerstick testing.

A preliminary clinical investigation conducted in 2024 demonstrated the technical feasibility of the system, reporting a mean absolute relative difference (MARD) of 20.3% and 97.3% of measurements within consensus error grid (CEG) zones A + B.^7^ However, that study included a heterogeneous population, had a short monitoring duration, and did not evaluate temporal changes in performance under extended real-world conditions.

This study was designed to address these limitations through a prospective clinical investigation evaluating the analytical performance, safety, and usability of GLUCUBE during 21 days of structured home use in adults with T2D. The primary objective was to assess measurement accuracy compared with a capillary blood glucose monitoring system with established analytical performance using MARD, Parkes CEG,^8^ and DTS error grid analyses.^9^ Secondary objectives included evaluation of temporal changes in performance, user adherence and satisfaction, and device safety under real-world ambulatory conditions.

## Methods

The study was conducted in accordance with the Declaration of Helsinki, ISO 14155:2020, and applicable regulatory requirements. The protocol was approved by the Institutional Ethics Committee of Seville (2024-NIGM-004) and by the Spanish Agency for Medicines and Medical Devices (AEMPS). The study was registered at ClinicalTrials.gov (NCT07075796), and all participants provided written informed consent before enrollment.

### Description of the Investigational Device (GLUCUBE)

GLUCUBE (Model ES0201, IGLUCO TECH, S.L., Seville, Spain) is a noninvasive glucose monitoring device based on NIRS that estimates glucose levels through finger transillumination. For each measurement, the participant placed a finger inside the device, where near-infrared light was transmitted through the fingertip. The transmitted optical signal was detected by internal photodetectors and processed using proprietary signal-processing algorithms designed to reduce optical noise and inter-individual variability. Glucose estimation was obtained through an individualized calibration procedure that adjusts the parameters of the glucose estimation model for each user following an initial calibration protocol requiring paired capillary glucose measurements. The model is initialized using predefined reference parameters and incorporates demographic and anthropometric variables (age, sex, height, and weight) as covariates. Individualization is achieved by automatically optimizing the model parameters using each participant’s paired capillary reference measurements, thereby improving glucose estimation accuracy for that specific user. The detailed mathematical implementation of the calibration algorithm is proprietary and cannot be disclosed. The final glucose estimate was displayed through a dedicated smartphone application. At the time this clinical investigation was conducted, GLUCUBE ES0201 was an investigational device and had not yet obtained CE marking. This study formed part of its pre-market clinical evaluation. Following completion of the clinical investigation and the regulatory assessment process, the device subsequently obtained CE marking and is currently commercially available. According to its current CE-marked intended use, GLUCUBE is designed as a noninvasive glucose monitoring device to support glucose follow-up and is not intended to guide insulin dosing decisions.

The overall architecture of the GLUCUBE system, including the measurement unit, smartphone application, and cloud-based processing workflow, is shown in Figure 1.

**Figure 1.**
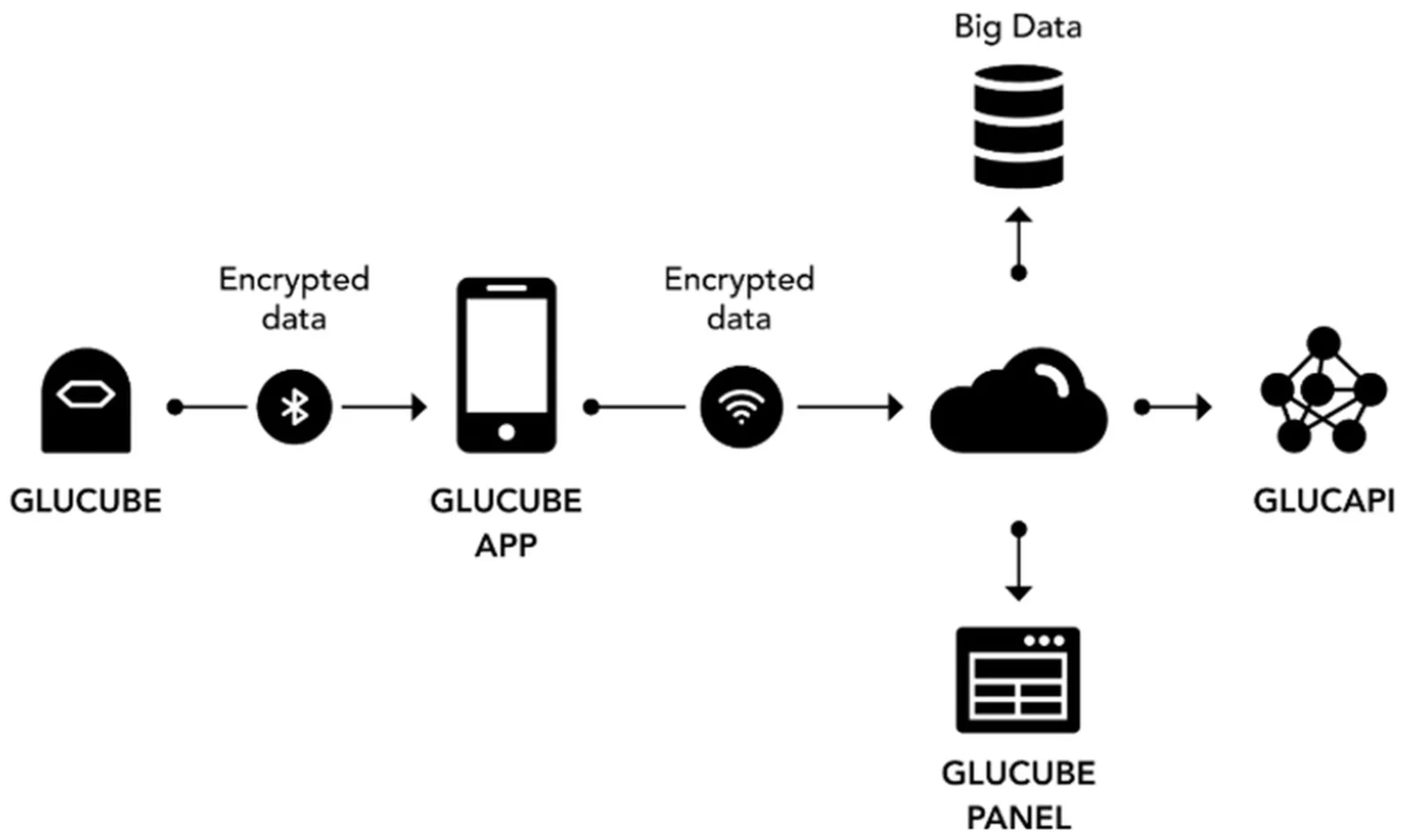
GLUCUBE system architecture, including the measurement unit, mobile application, and cloud-based processing module.

### Description of the Reference Device (Ascensia Contour Next)

Reference glucose measurements were obtained using the Ascensia Contour Next glucometer (Ascensia Diabetes Care, Parsippany, NJ, USA), a capillary blood glucose monitoring system compliant with ISO 15197:2013 accuracy standards.^10^ The device was selected because of its well-established analytical performance and widespread clinical use. Laboratory reference methods such as YSI were not incorporated because the objective was to evaluate performance under real-world ambulatory conditions.^11^

### Study Population

Eligible participants were adults (≥18 years) with T2D who owned a compatible smartphone and were able to operate the study device independently. Key exclusion criteria included pregnancy, severe glycemic instability (defined as recent episodes of severe hypoglycemia or acute metabolic decompensation, as assessed by the investigator during screening), and participation in another clinical investigation. Participants with a reference glucose coefficient of variation (CV) >36% were excluded from the Per-Protocol Set (PPS) for the primary performance analysis, as prespecified in the statistical analysis plan. This threshold was used to identify participants with extreme glucose variability, who were considered not representative of the target user population because excessive physiological glucose variability could artificially inflate device error metrics and misrepresent typical device performance. These participants remained in the Study Completers Set (SSC) and were included in the predefined sensitivity analyses.^12^ The study was restricted to adults with T2D because this population represents the intended target population for the current stage of device development.

### Study Design

This was a prospective, single-center, open-label clinical investigation conducted between May and November 2025 at Hospital Universitario Virgen del Rocío (Seville, Spain). Fifty-two adults with T2D were enrolled. Participants were able to view both the glucose estimates generated by GLUCUBE and the paired reference capillary glucose measurements throughout the study. Participants continued their usual diabetes treatment throughout the study, and no protocol-mandated changes to diabetes therapy were made. GLUCUBE measurements were not used to guide therapeutic decisions, and all treatment decisions continued to rely on conventional capillary glucose measurements according to usual clinical practice.

At visit 1 (day 1), participants underwent eligibility screening, provided written informed consent, and received standardized training on the use of GLUCUBE and the reference glucometer. Baseline demographic and clinical data were collected.

Participants were instructed to perform 4 paired glucose measurements daily for 21 days at standardized time points (fasting, preprandial before lunch, 2-h postprandial after lunch, and bedtime), consistent with previously described paired glucose testing methodologies.^13^ For each paired measurement, participants first obtained a noninvasive glucose estimation using GLUCUBE followed immediately by capillary glucose measurement using the reference glucometer. Reference values were manually entered into the GLUCUBE application to enable paired data recording and subsequent accuracy analysis. According to the device instructions for use, GLUCUBE measurements could be performed on any finger of either hand except the thumb and little finger, with the ring finger being the recommended measurement site. The specific finger used for each measurement was left to the participant’s usual practice and was not recorded. Capillary glucose sampling followed each participant’s usual practice, and the study protocol did not require the finger used for GLUCUBE measurements to be excluded from capillary sampling. No standardized glucose challenges, meal tests, or experimentally induced glycemic excursions were performed, as the study was designed to evaluate device performance under routine real-world conditions.

At visit 2 (day 22 ± 2 days), participants returned for final evaluation, including assessment of usability, satisfaction, and adverse events. Reference glucometer values stored in device memory were cross-checked against manually entered values in the GLUCUBE application. All study data were managed using an electronic data capture system (OpenClinica, Version 3.13; OpenClinica LLC, Waltham, MA, USA).

### Primary Performance Analysis

Analytical performance was evaluated using a combined MAD/MARD approach, together with Parkes CEG and DTS error grid analyses. For each paired measurement, analytical error was calculated as the mean absolute difference (MAD, mg/dL) when the reference glucose value was <100 mg/dL and as the MARD (%) when the reference glucose value was ≥100 mg/dL, consistent with ISO 15197:2013.^10^ The overall combined MAD/MARD value was obtained by applying the appropriate error metric to each paired measurement according to this predefined 100 mg/dL threshold. In addition, the MAD and MARD components were reported separately according to the reference glucose range, together with the proportions of measurements within ±20, ±30, and ±40 mg/dL for reference values <100 mg/dL and within ±20%, ±30%, and ±40% for reference values ≥100 mg/dL.

As a secondary performance analysis, analytical accuracy was also evaluated according to clinically relevant reference glucose ranges (<70, 70-180, and >180 mg/dL). For each range, the number and proportion of paired measurements, the corresponding accuracy metric (MAD or MARD, as appropriate), and the proportion of measurements within the predefined ±20 mg/dL or ±20% accuracy threshold were reported.

### Statistical Analysis

Several predefined analysis populations were used, including the Safety Analysis Set (SAS), SCS, PPS, Acceptable Adherence Population (AAP), and High-Adherence Population (HAP). Continuous variables are presented as mean ± SD or median (interquartile range [IQR]), and categorical variables are presented as frequencies and percentages. Accuracy was assessed using the combined MAD/MARD metric, Parkes CEG, and DTS error grid analyses. The MAD and MARD components were also reported separately according to the predefined reference glucose threshold (<100 and ≥100 mg/dL, respectively). Temporal changes in performance were evaluated using repeated-measures analysis of variance or nonparametric equivalents, as appropriate, with Bonferroni-adjusted pairwise comparisons when significant. Sensitivity analyses compared PPS and SCS populations. Missing data were handled using complete-case analysis without imputation. Data processing and statistical analyses were conducted jointly by the clinical investigators and technical personnel from IGLUCO TECH, S.L., using the complete paired dataset obtained from the investigational device and the reference capillary glucose measurements. Statistical significance was defined as *P* < .05.

### User Satisfaction and Safety

User satisfaction was assessed at visit 2 using a structured 5-item questionnaire addressing ease of use, perceived usefulness, preference compared with capillary monitoring, perceived impact on monitoring frequency, and overall satisfaction. Responses were recorded using 5-point Likert scales and summarized descriptively.

Safety was assessed throughout the study by monitoring adverse events, serious adverse events, and device-related complications. Safety outcomes were analyzed descriptively in the SAS.

## Results

### Study Population and Participant Disposition

Between May and November 2025, a total of 52 participants were enrolled and provided written informed consent. Two participants (3.8%) withdrew consent during the home monitoring phase for personal reasons unrelated to device safety or performance, resulting in 50 participants (96.2%) who completed the study.

Of the study completers, 2 participants (4.0%) were excluded from the PPS due to extreme glucose variability (CV >36% in reference measurements) but were retained in the SCS for sensitivity analyses. The final PPS comprised 48 participants (92.3% of enrolled), who were included in the primary accuracy analysis.

Among PPS participants, 39 (81.3%) met the criteria for the AAP, and 33 (68.8%) met the criteria for the HAP. The SAS included all 52 enrolled participants. Participant disposition is summarized in Figure 2.

**Figure 2.**
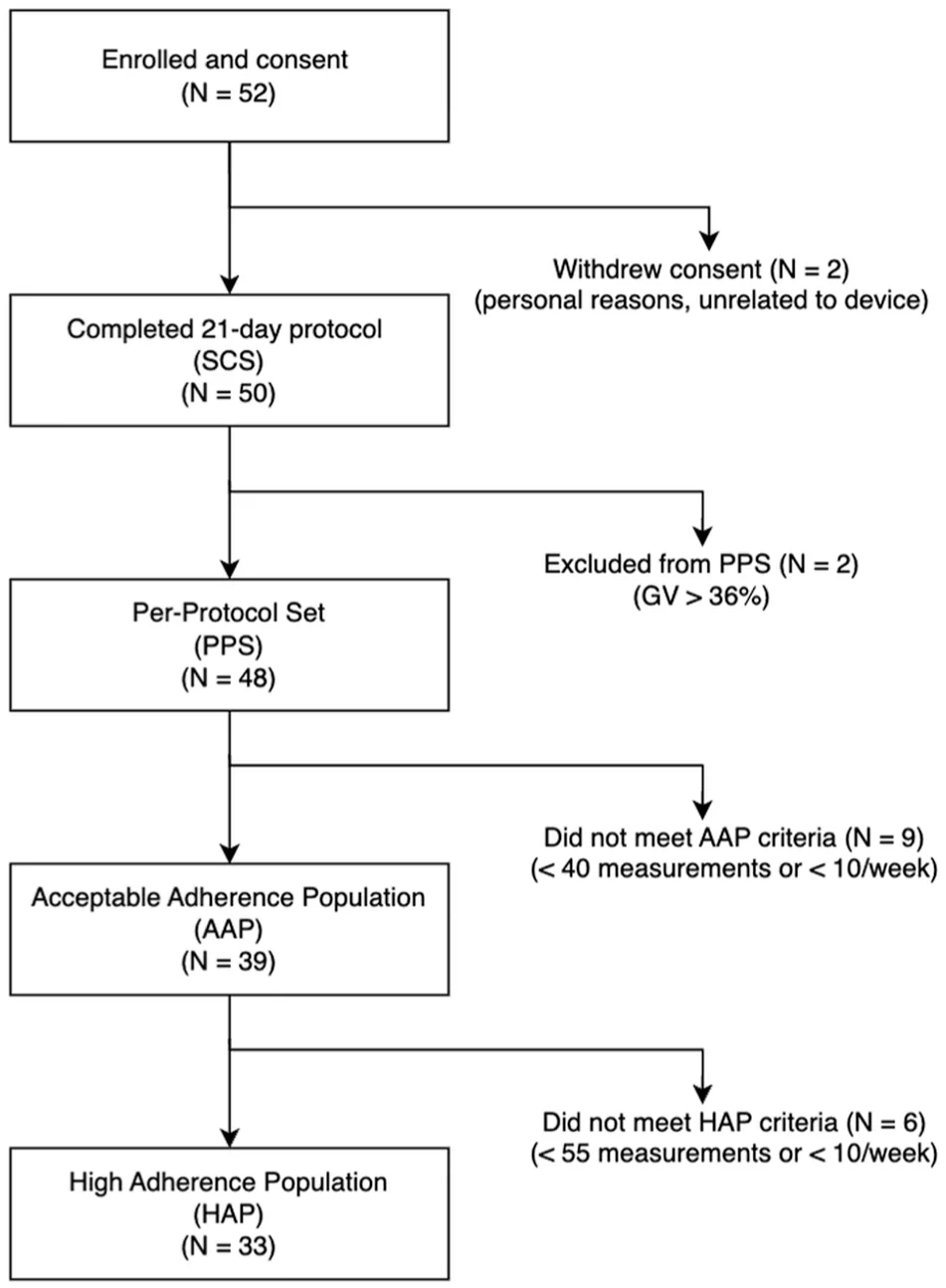
Flow diagram of participant disposition, including enrollment, study completion, derivation of analysis populations (SAS, SCS, PPS, AAP, and HAP), and reasons for exclusion at each stage.

### Safety

No serious adverse events (SAEs), device-related adverse events (AEs), or study discontinuations due to safety concerns were reported during the 21-day monitoring period. All study procedures were well tolerated.

### Baseline Characteristics

Baseline demographic and clinical characteristics of the study population are presented in Table 1. Overall, participants were predominantly male (71.2% in SAS; 68.8% in PPS), with a mean age of 58.0 years and a mean body mass index (BMI) of approximately 31 kg/m^2^, consistent with an overweight/obese T2D population. Mean HbA1c was 7.5% in the SAS and 7.4% in the PPS, indicating moderately controlled diabetes.

**Table 1.** Baseline Demographic and Clinical Characteristics.

	SAS (n = 52)	PPS (n = 48)
Sex, n (%)		
Male	37 (71.2)	33 (68.8)
Female	15 (28.8)	15 (31.3)
Race, n (%)		
Caucasian	51 (98.1)	47 (97.9)
Andean	1 (1.9)	1 (2.1)
Education level, n (%)		
Primary education	17 (32.7)	14 (29.2)
Secondary education	23 (44.2)	23 (47.9)
University degree	11 (21.2)	10 (20.8)
Postgraduate degree	1 (1.9)	1 (2.1)
Income level, n (%)		
Low (<11,000 €)	5 (9.6)	5 (10.4)
Middle (11,000- 30,000 €)	43 (82.7)	39 (81.3)
High (>30,000 €)	4 (7.7)	4 (8.3)
Age, years	58.0 ± 9.7 (33-78)	58.0 ± 9.9 (33-78)
Height, m	1.71 ± 0.09 (1.53-1.89)	1.71 ± 0.09 (1.53-1.89)
Weight, kg	92.0 ± 19.0 (52-137.4)	91.9 ± 18.8 (52-137.4)
BMI, kg/m^2^	31.3 ± 6.6 (18.4-49.2)	31.6 ± 6.6 (18.4-49.2)
HbA1c, %	7.5 ± 1.8 (4.8-15.4)	7.4 ± 1.8 (4.8-15.4)
Antidiabetic therapy, n (%)		
Insulin therapy	18 (34.6)	15 (31.3)
Non-insulin glucose-lowering medication	50 (96.2)	47 (97.9)
Systolic blood pressure, mm Hg	132.8 ± 20.8 (82-192)	134.1 ± 10.1 (82-192)
Diastolic blood pressure, mm Hg	86.7 ± 13.1 (60-136)	87.0 ± 13.3 (60-136)

BMI, body mass index; HbA1c, glycated hemoglobin; SAS, Safety Analysis Set; PPS, Per-Protocol Set.

No relevant differences were observed between the SAS and PPS populations, supporting the representativeness of the primary analysis cohort.

### Glucose Dataset

Over the 21-day monitoring period, participants in the PPS (n = 48) generated a total of 3,093 paired GLUCUBE–reference glucose measurements. Of these, 3,085 measurements (99.7%) had reference glucose values within the prespecified analysis range of 70 to 400 mg/dL and were included in the primary performance analysis. Eight paired measurements (0.3%) with reference glucose values outside the predefined analytical range (70-400 mg/dL) were excluded from the primary analysis. All excluded reference glucose values were between 60 and 69 mg/dL (level 1 hypoglycemia), and none represented level 2 hypoglycemia (<54 mg/dL). Three of these measurements occurred during the initial calibration period. In 5 paired measurements, GLUCUBE yielded a normal or elevated glucose value despite a hypoglycemic reference measurement, whereas the remaining 3 showed only small differences between the reference and device measurements.

Among the included measurements, 469 corresponded to calibration data (initial paired measurements used for device calibration), whereas 2,616 represented post-calibration measurements used for accuracy assessment. Reference glucose values ranged from 71 to 372 mg/dL, with a mean of 141.4 ± 44.6 mg/dL and a median of 131 mg/dL. The distribution of reference glucose values is presented in Figure 3.

**Figure 3.**
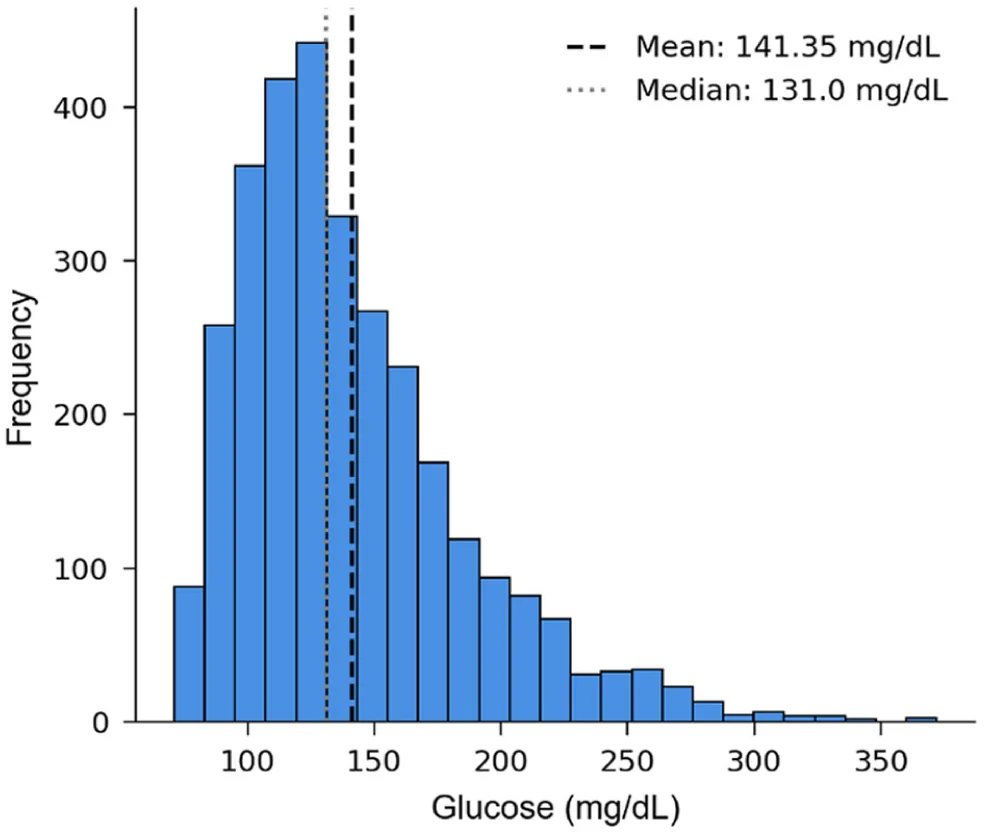
Distribution of reference glucose values (mg/dL) across all paired measurements included in the primary analysis.

### Primary Performance Analysis

The primary performance analysis was conducted using 2,616 post-calibration paired measurements obtained from 48 participants in the PPS, excluding the initial calibration measurements.

Parkes CEG analysis demonstrated 69.9% of measurements in zone A, 29.1% in zone B, 1.0% in zone C, and no measurements in zones D or E (Figure 4). The complementary DTS error grid analysis is provided in Supplemental Figure S1.

**Figure 4.**
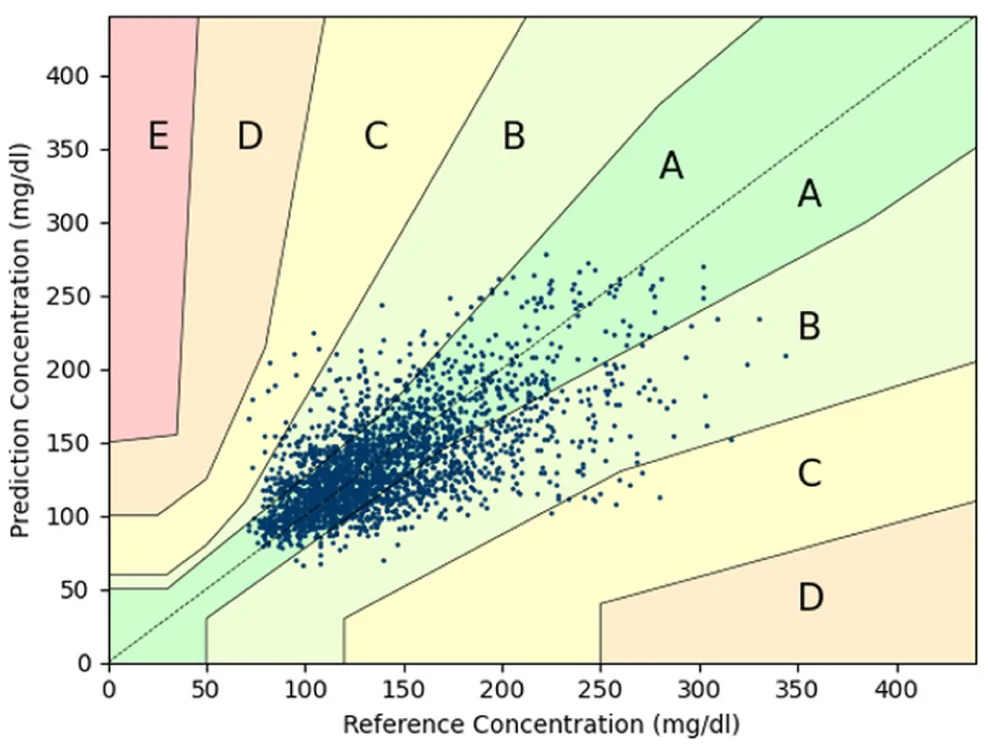
Parkes consensus error grid analysis of 2,616 post-calibration paired GLUCUBE–reference glucose measurements showing the distribution of values across clinical risk zones.

The overall combined MAD/MARD value was 16.3, calculated by applying absolute error to paired measurements with reference glucose values <100 mg/dL and relative error to those with reference glucose values ≥100 mg/dL, in accordance with ISO 15197:2013.^10^ Of the 2,616 post-calibration paired measurements, 379 (14.5%) had reference glucose values <100 mg/dL and were evaluated using MAD, whereas 2,237 (85.5%) had reference glucose values ≥100 mg/dL and were evaluated using MARD (Table 2).

**Table 2.** Combined MAD/MARD Analytical Performance According to Reference Glucose Range.

Reference glucose range (mg/dL)	n (%)	Accuracy metric	Value	Within ±20	Within ±30	Within ±40
<100	379 (14.5)	MAD	19.4 mg/dL	66.2%	79.4%	89.7%
≥100	2,237 (85.5)	MARD	15.8%	70.5%	87.4%	95.3%

Combined MAD/MARD was calculated on a point-by-point basis by applying MAD to paired measurements with reference glucose values <100 mg/dL and MARD to paired measurements with reference glucose values ≥100 mg/dL.

The proportion of measurements meeting predefined accuracy thresholds was 69.8% within ±20 mg/dL or ±20%, 86.3% within ±30 mg/dL or ±30%, and 94.5% within ±40 mg/dL or ±40% of reference values.

As a secondary analysis, analytical performance was also evaluated according to clinically relevant reference glucose ranges (Table 3). No post-calibration paired measurements with reference glucose values <70 mg/dL were available because the predefined analytical dataset included only measurements within the 70 to 400 mg/dL range. Within the target glucose range (70-180 mg/dL), which comprised 2,204 paired measurements (84.3%), the combined MAD/MARD value was 15.4, with 73.6% of measurements meeting the predefined ±20 mg/dL or ±20% accuracy criterion. This included 379 paired measurements between 70 and <100 mg/dL (MAD: 19.4 mg/dL; 66.2% within ±20 mg/dL) and 1,825 paired measurements between 100 and 180 mg/dL (MARD: 14.5%; 75.1% within ±20%). For reference glucose values >180 mg/dL (n = 412; 15.8%), the MARD was 21.4%, with 49.8% of measurements within ±20%.

**Table 3.** Secondary Analytical Performance According to Clinically Relevant Reference Glucose Ranges.

Reference glucose range (mg/dL)	Clinical category	n (%)	Accuracy metric	Value	Measurements within predefined accuracy criterion
<70	Hypoglycemia	0 (0)	—	—	—
70 to 180	Target range	2,204 (84.3)	Combined MAD/ MARD	15.4	73.6% within ±20 mg/dL or ±20%*
70 to <100		379	MAD	19.4 mg/dL	66.2% within ±20 mg/dL
100 to 180		1,825	MARD	14.5%	75.1% within ±20%
>180	Hyperglycemia	412 (15.8)	MARD	21.4%	49.8% within ±20%

MAD, mean absolute difference; MARD, mean absolute relative difference.

* Combined MAD/MARD was calculated by applying the MAD to paired measurements with reference glucose values <100 mg/dL and the MARD to paired measurements with reference glucose values ≥100 mg/dL.

*Note.* No post-calibration paired measurements with reference glucose values <70 mg/dL were available because the primary post-calibration analytical dataset was restricted to the predefined reference glucose range of 70 to 400 mg/dL.

During the initial calibration phase (n = 469 measurements), analytical performance was slightly lower than during the post-calibration period, consistent with the establishment of individualized calibration parameters and/or increasing user familiarity with the measurement procedure. When calibration measurements were included, overall, MARD was 16.8%, with 98.9% of values within CEG zones A + B. These measurements were excluded from the primary analysis, as they represent a transitional phase rather than steady-state device performance. Following calibration, performance stabilized and remained consistent throughout the remainder of the study period.

### Sensitivity Analysis

Sensitivity analyses showed minimal differences between PPS and SCS populations (MARD 16.9% vs 16.3%; CEG zones A + B 98.7% vs 99.0%), supporting the robustness of the primary analysis.

### Performance Over Time (Acceptable Adherence Population)

Of the 48 participants included in the PPS, 39 (81.3%) met the criteria for the AAP, contributing a total of 2,812 paired measurements for temporal analysis. Measurements were distributed across the study period as follows: week 1 (days 1-7, n = 939), week 2 (days 8-14, n = 913), and week 3 (days 15-21, n = 860).

Weekly performance metrics are summarized in Table 4. MARD decreased from 18.7% in week 1 to 16.2% in week 2 and 16.0% in week 3. Consensus error grid performance improved from 98.2% in week 1 to 99.2% in week 2 and 99.3% in week 3. Similarly, the proportion of measurements within ±20% increased from 63.2% (week 1) to 71.1% (week 2) and 69.7% (week 3), while ±30% and ±40% thresholds showed consistent improvement. The distribution of individual participant MARD values across the 3 weeks is shown in Figure 5A.

**Table 4.** Weekly Analytical Performance of GLUCUBE in the Acceptable Adherence Population (AAP) and High-Adherence Population (HAP).

Population	Week	N measurements	Combined MAD/MARD	Zones A + B (%)	Within ±20%	Within ±30%	Within ±40%
AAP	Week 1	939	18.7	98.2	63.2	80.9	90.6
	Week 2	913	16.2	99.2	71.1	85.7	94.3
	Week 3	860	16.0	99.3	69.7	87.0	95.8
HAP	Week 1	832	18.9	98.1	63.0	80.7	90.5
	Week 2	829	16.5	99.2	70.6	85.4	94.1
	Week 3	776	16.5	99.2	68.0	86.3	95.6

AAP, Acceptable Adherence Population (≥40 paired measurements and ≥10 measurements/week); HAP, High-Adherence Population (≥55 paired measurements and ≥10 measurements/week); MAD, mean absolute difference; MARD, mean absolute relative difference.

Combined MAD/MARD was calculated on a point-by-point basis using MAD for reference glucose values <100 mg/dL and MARD for values ≥100 mg/dL.

**Figure 5.**
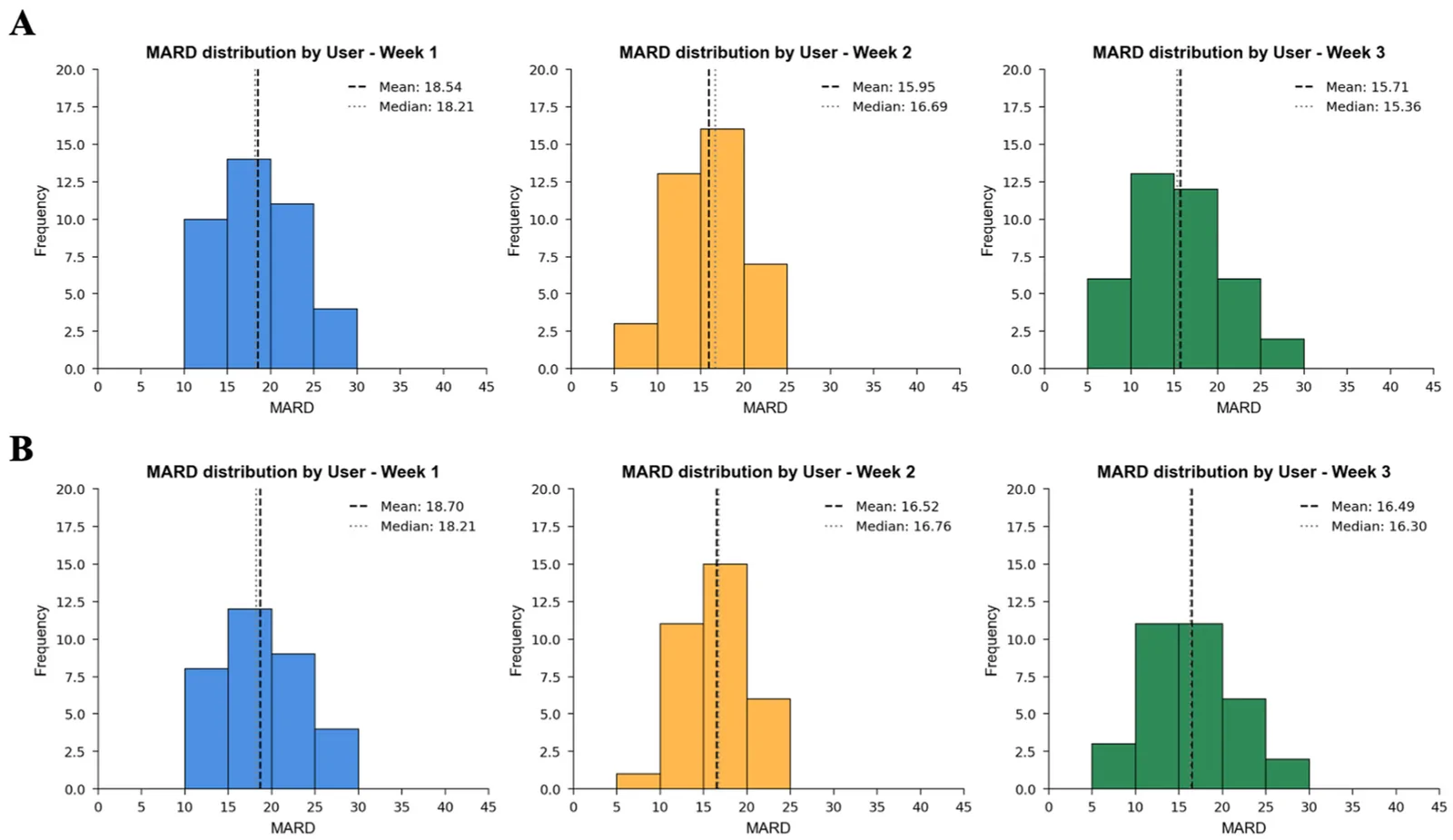
Temporal evolution of individual participant MARD values across study weeks in adherence-defined populations. (A) Acceptable Adherence Population (AAP). (B) High-Adherence Population (HAP). MARD, mean absolute relative difference.

Repeated-measures analysis of variance demonstrated a significant effect of time on participant-level MARD (Figure 5A). Mean participant-level MARD decreased from 18.5% (SD: 4.7%) in week 1 to 16.0% (SD: 4.3%) in week 2 and 15.7% (SD: 5.3%) in week 3, *F*(2,76) = 12.31, *P* < .001, η^2^G = 0.069.

Post hoc comparisons using Bonferroni correction demonstrated a significant reduction in MARD between week 1 and week 2 (*P* <.001) and between week 1 and week 3 (*P* < .001), whereas no significant difference was observed between weeks 2 and 3 (*P* = 1.000). Overall, these findings indicate a reduction in measurement error after the first week of use, followed by stabilization of performance during the remainder of the study period.

### Performance Over Time (High-Adherence Population)

Of the 48 participants included in the PPS, 33 (68.8%) met the criteria for the HAP, contributing a total of 2,437 paired measurements for temporal analysis. Measurements were distributed as follows: week 1 (days 1-7, n = 832), week 2 (days 8-14, n = 829), and week 3 (days 15-21, n = 776).

Weekly performance metrics are summarized in Table 4. MARD decreased from 18.9% in week 1 to 16.5% in weeks 2 and 3. The proportion of measurements within CEG zones A + B increased from 98.1% in week 1 to 99.2% in weeks 2 and 3. Similarly, the proportion of measurements within ±20% of reference values increased from 62.3% in week 1 to 70.6% in week 2 and remained stable at 68% in week 3, with similar improvements observed for the ±30% and ±40% accuracy thresholds.

The distribution of individual participant MARD values across study weeks is shown in Figure 5B. Repeated-measures analysis of variance demonstrated a significant effect of time on participant-level MARD, *F*(2,64) = 8.11, *P* < .001, η^2^G = 0.051. Mean participant-level MARD values were 18.7% (SD: 4.6%) in week 1, 16.5% (SD: 3.7%) in week 2, and 16.5% (SD: 5.2%) in week 3.

Post hoc pairwise comparisons with Bonferroni correction showed significantly lower MARD values in weeks 2 (*P* = .003) and 3 (*P* = .008) compared with week 1, whereas no significant difference was observed between weeks 2 and 3 (*P* = 1.000).

### Exploratory Analysis of Glycemic Changes During Monitoring

To explore potential changes in glycemic control during the study period, mean participant-level glucose values were compared between week 1 (baseline) and week 3 in the HAP (n = 33).

Mean glucose decreased from 144.7 ± 36.8 mg/dL in week 1 to 138.9 ± 31.0 mg/dL in week 3, corresponding to a mean reduction of 5.8 mg/dL per participant. Paired differences were normally distributed (Shapiro-Wilk *P* = .076). The reduction was statistically significant (paired t-test, *P* = .016; 95% CI [confidence interval]: −10.4 to −1.2 mg/dL; Cohen’s d = 0.44). Given the exploratory nature of the analysis, the absence of a control group, and the single-arm study design, these findings should be interpreted cautiously and considered hypothesis-generating.

### Safety

No AEs or SAEs were reported during the 21-day study period. All participants completed the study without device-related complications, including local skin reactions, discomfort, pain, bleeding, or infection at measurement sites. No device malfunctions or deficiencies affecting safety were identified.

### User Satisfaction

User satisfaction was assessed in 50 of 52 participants (96.2%) who completed a 5-item questionnaire at visit 2. Responses were recorded on 5-point Likert scales. The highest-rated domain was ease of use (mean: 4.3 ± 1.2). Other domains showed moderate scores, including preference compared with capillary monitoring (mean: 2.9 ± 1.5; median: 3.0), perceived usefulness for glucose control (mean: 3.0 ± 1.4; median: 3.0), perceived impact on monitoring frequency (mean: 3.3 ± 1.4; median: 4.0), and overall satisfaction (mean: 3.1 ± 1.4; median: 3.0).

## Discussion

This study evaluated the analytical performance, safety, and usability of GLUCUBE, a near-infrared spectroscopy–based noninvasive glucose monitoring device, in adults with T2D under real-world ambulatory conditions. Overall, the findings support the technical feasibility and clinical potential of this investigational approach, demonstrating clinically relevant glucose estimation, stable performance following individualized calibration, and a favorable safety profile.

The Parkes CEG provides clinically relevant context for the observed glucose estimates, with 69.9% of measurements classified in zone A, 29.1% in zone B, 1.0% in zone C, and none in zones D or E. The absence of measurements in the highest-risk zones indicates a low likelihood of clinically significant treatment errors. Nevertheless, the small proportion of measurements classified in zone C should be interpreted in the context of the device’s intended use. GLUCUBE is intended as a noninvasive glucose monitoring device to support glucose follow-up and is not intended to guide insulin dosing decisions. Therefore, the clinical implications of occasional zone C measurements should be interpreted within the device’s intended-use framework rather than that of devices intended for direct therapeutic decision-making, such as insulin dose adjustment.

Although methodological differences exist in the calculation and reporting of accuracy metrics across studies, the reported values provide a useful contextual benchmark. In comparison with contemporary CGM systems, such as FreeStyle Libre 2 (reported overall combined MAD/MARD 9.2%)^14^ and Dexcom G6 (reported overall MARD 9%),^15^ the device showed lower analytical precision; however, this difference should be interpreted in the context of its fully noninvasive design and intended clinical role. While CGM use continues to increase, standard capillary SMBG remains the most widely used glucose monitoring method in many people with diabetes. Therefore, the potential clinical role of a noninvasive monitoring device should be considered in relation to both monitoring strategies. Continuous glucose monitoring systems require subcutaneous sensor insertion, periodic replacement, and ongoing skin contact, which may limit acceptability in certain patient populations. In contrast, the device eliminates routine skin penetration after calibration, offering a fundamentally different value proposition. Importantly, in populations with low baseline adherence to glucose monitoring, the clinical value of a noninvasive monitoring approach may extend beyond analytical performance alone and could depend on factors such as user acceptance, monitoring frequency, and long-term engagement.

Rather than competing directly with CGM systems, the device may expand access to glucose monitoring among individuals who currently do not use any monitoring strategy. In this context, a noninvasive monitoring approach may help address barriers to glucose monitoring in selected individuals with T2D.

Relative to previously reported noninvasive glucose monitoring approaches, the present findings suggest clinically meaningful progress. Optical-based systems, including mid-infrared spectroscopy platforms, have historically demonstrated MARD values in the range of 19% to 21%.^16^ The analytical performance observed in this study, together with the favorable error grid agreement, suggests that individualized calibration and signal-processing approaches may contribute to improved performance in noninvasive monitoring systems. Unlike many previous NIRS-based approaches that relied predominantly on optical measurements alone, GLUCUBE integrates individualized calibration based on paired reference measurements together with participant-specific physiological variables and advanced signal-processing techniques. Although this study was not designed to directly compare different NIRS technologies, this multimodal approach was specifically developed to address major sources of inter-individual variability that have historically limited the clinical performance of noninvasive optical glucose monitoring.

This study also extends prior feasibility data reported for this device.^7^ Compared with earlier investigations conducted over shorter durations and in more heterogeneous populations, this study incorporated a longer ambulatory period, a broader glycemic range, and a structured calibration protocol. The observed improvement in performance is consistent with the potential contribution of iterative calibration and real-world use conditions to optimizing measurement accuracy in noninvasive systems.^7^ However, comparisons across studies should be interpreted cautiously because of differences in study populations, reference methods, monitoring duration, and analytical methodologies.

A relevant observation was the temporal evolution of accuracy during the study period. Measurement error decreased significantly after the first week of use and remained stable thereafter, a pattern consistently observed across adherence-defined populations. This 2-phase pattern, characterized by an initial improvement followed by stabilization, is consistent with the combined effects of individualized calibration and increasing user familiarity with the device. The marked improvement observed after exclusion of the initial calibration phase suggests that individualized calibration was likely the principal contributor. In addition, an independent analysis of the AAP demonstrated improved measurement acceptance after the first week of use in 24 of 39 participants (61.5%), supporting a possible learning effect in device handling. However, calibration parameters and user technique metrics were not jointly modeled; therefore, the relative contribution of these 2 mechanisms cannot be quantified from the present data. These findings underscore the importance of both individualized calibration and appropriate user familiarization during the initial period of device use. From a practical perspective, performance observed after the first week may more accurately reflect steady-state use in routine clinical settings. Although the overall improvement in combined MAD/MARD after incorporation of individualized calibration data was modest (16.8% vs 16.3%), the temporal analysis demonstrated a more pronounced improvement during the first week of use, followed by stable performance in weeks 2 and 3. These findings suggest that most of the improvement in analytical performance occurs during the initial adaptation period, after which device performance remains stable. Although individualized calibration appears to play a major role in this improvement, the contribution of increasing user familiarity cannot be excluded and warrants further investigation.

A modest reduction in mean glucose levels was observed in the high-adherence subgroup over the study period. Although statistically significant, this finding should be interpreted cautiously. The single-arm design, short follow-up duration, and absence of a control group preclude causal inference. The observed change may reflect increased patient engagement, behavioral adaptation, or regression to the mean, rather than a direct effect of the device itself. In addition, the mandatory frequent paired capillary glucose measurements required by the study protocol may have increased participants’ awareness of their glucose levels and influenced glycemic behavior independently of any device-specific effect (Hawthorne effect). Nonetheless, these findings are consistent with prior evidence suggesting that more frequent glucose monitoring may enhance self-awareness and support behavioral adjustments in individuals with diabetes. Future studies performed under routine clinical use conditions, ideally including baseline self-monitoring behavior, will be needed to better distinguish the independent effect of the device from that of increased monitoring frequency.

This study was conducted in adults with T2D receiving routine clinical care, a population in whom extreme glycemic excursions and hypoglycemic events are generally less frequent than in insulin-treated type 1 diabetes (T1D). Consequently, the evaluated glucose range may not fully represent more unstable glycemic profiles. Further studies including individuals with greater glycemic variability and more frequent hypoglycemia are warranted to further characterize device performance across the full spectrum of glucose concentrations.

From a clinical perspective, the device may be particularly relevant for individuals with T2D who perform infrequent or no regular glucose monitoring despite existing recommendations. Barriers to current monitoring strategies include discomfort associated with capillary testing, reluctance to use invasive sensors, perceived device burden, and practical constraints in daily life. In this context, the device may reduce barriers to engagement with glucose monitoring and could facilitate more frequent monitoring in some individuals with T2D.

Accordingly, the device is best conceptualized not only as a replacement for high-accuracy CGM systems in intensively managed populations but also as a complementary monitoring approach for individuals who do not routinely use existing monitoring technologies. Its potential clinical value may, therefore, lie less in maximizing analytical precision and more in facilitating access to glucose information in previously under-monitored populations. Further studies are required to determine whether this approach can improve long-term monitoring engagement and clinical outcomes.

The requirement for an initial calibration phase represents a current technical limitation; however, it enables individualized calibration, which likely contributes to the observed accuracy improvements. Although this necessitates a short period of capillary testing, it substantially reduces the long-term burden of fingerstick measurements compared with conventional self-monitoring strategies. Further technological refinements aimed at reducing or simplifying calibration may enhance usability and facilitate broader adoption.

User satisfaction results were moderate and should be interpreted within the context of the study design. Although participants rated the device as easy to use, overall satisfaction and preference over conventional capillary monitoring were more modest. This finding should be interpreted in the context of the study design, which required paired capillary fingerstick measurements throughout the study despite evaluating a device intended to reduce or avoid routine fingersticks. Consequently, participants were unable to fully experience one of the device’s principal intended advantages, which may have contributed to the moderate satisfaction scores. The gradual decline in the number of measurements over the 3-week study period, despite participants meeting the predefined adherence thresholds, may reflect the practical challenges of sustaining frequent daily measurements under the study protocol. This observation is consistent with the moderate user satisfaction scores and further supports the need for studies evaluating long-term engagement under routine post-calibration conditions. Future studies incorporating post-calibration real-world use conditions will be important to more accurately capture user acceptance and real-world utility.

This study has several strengths. It represents, to our knowledge, one of the first ambulatory evaluations with more than 2,600 post-calibration paired measurements of a near-infrared spectroscopy–based noninvasive glucose monitoring device conducted under real-world home-use conditions. The study design included a structured calibration protocol, a large number of paired measurements, and longitudinal assessment of performance beyond the initial calibration phase, allowing evaluation of both calibration effects and subsequent performance stability under routine home-use conditions.

Despite these encouraging findings, the analytical performance of GLUCUBE should be interpreted in the context of its current developmental stage. Although the observed analytical performance supports the clinical feasibility of this NIRS-based approach, it remains below the performance achieved by currently available minimally invasive CGM systems. Furthermore, the need for individualized calibration and the evaluation in a relatively stable T2D population indicate that additional optimization and validation are required before broader clinical implementation. Future studies should assess device performance across more diverse populations, wider glycemic ranges, and longer follow-up periods.

Several limitations should be acknowledged. The study was conducted at a single center, included a moderate sample size, and had a relatively short duration. The absence of a control group limits conclusions regarding clinical effectiveness. Performance was evaluated within a predefined glucose range (70-400 mg/dL), and extrapolation beyond this range is not supported. Although measurements with reference glucose values <70 mg/dL were excluded from the predefined primary accuracy analysis, inspection of these excluded paired measurements indicates the need for further evaluation of device performance at low glucose concentrations. Future studies specifically designed to include a greater number of hypoglycemic measurements will be important to better characterize device performance in this clinically relevant range. The study was restricted to adults with T2D, and therefore, the findings cannot be generalized to individuals with T1D. No standardized glucose challenges or experimentally induced glycemic excursions were performed, limiting assessment under rapidly changing glycemic conditions. In addition, paired measurements were obtained at 4 predefined daily time points, including only 1 postprandial period. Although this standardized approach reduced variability between participants, it may not fully represent the broader range of glycemic conditions encountered during unrestricted real-world device use. Furthermore, glucose measurements were compared with a validated capillary glucose monitoring system rather than laboratory-based reference methods such as YSI or radiometer analyzers. In addition, the study population was almost exclusively Caucasian, and Fitzpatrick skin type was not recorded. As optical NIRS measurements may be influenced by skin pigmentation, the generalizability of these findings to populations with a broader range of skin pigmentation and Fitzpatrick skin types remains uncertain and should be evaluated in future studies. Finally, the user-dependent nature of the device may introduce variability that could differ across populations and real-world settings. Potential interference from medications, endogenous chromophores, or other physiological factors affecting NIRS measurements was not specifically evaluated and warrants further investigation in future studies.

Future research should include longer-duration studies to assess calibration stability over time, determine the durability of individualized calibration beyond the 3-week study period, define optimal recalibration intervals, and evaluate real-world effectiveness. In particular, studies targeting individuals with low baseline monitoring adherence will be essential to determine whether noninvasive technologies can improve sustained engagement and translate into meaningful clinical outcomes.

## Conclusion

GLUCUBE demonstrated clinically relevant glucose estimation, a favorable safety profile, and stable performance under ambulatory conditions in adults with T2D. Although analytical precision remains lower than both current CGM systems and standard capillary blood glucose monitoring, its fully noninvasive design supports further development as a complementary glucose monitoring approach for individuals who are unwilling or unable to use existing invasive monitoring technologies. By reducing barriers associated with conventional glucose monitoring, GLUCUBE may facilitate access to glucose information in previously under-monitored populations. Further studies are warranted to evaluate long-term performance, optimize calibration strategies, and determine its impact on monitoring adherence and clinical outcomes.

## Supplemental Material

sj-docx-1-dst-10.1177_19322968261496268 – Supplemental material for Real-World Evaluation of a Near-Infrared Spectroscopy–based Noninvasive Glucose Monitoring Device in Adults With Type 2 Diabetes Supplemental material, sj-docx-1-dst-10.1177_19322968261496268 for Real-World Evaluation of a Near-Infrared Spectroscopy–based Noninvasive Glucose Monitoring Device in Adults With Type 2 Diabetes by Sandra Amuedo, Mónica Enríquez Macías, José Manuel Fornés, Arturo Moreno Arteaga, Jesús Riesgo Barrau, Virginia Bellido, Fernando Losada Viñau, Luz María Hidalgo Pichardo, Alfonso Soto Moreno, Antonio Puppo Moreno and Alfonso Pumar López in Journal of Diabetes Science and Technology

## Data Availability

The data supporting the findings of this study are available from the corresponding author upon reasonable request. The data are not publicly available due to privacy considerations and restrictions related to the funding agreement.*
