# Supplementary material for: Real-World Evaluation of a Near-Infrared Spectroscopy–based Noninvasive Glucose Monitoring Device in Adults With Type 2 Diabetes
Source: J Diabetes Sci Technol. 2026 Oct 25:19322968261496268. Online ahead of print. doi: 10.1177/19322968261496268 (PMC13696587; doi:10.1177/19322968261496268)
Supplement: Supplementary file 1 [file sj-docx-1-dst-10.1177_19322968261496268.docx]

**Supplementary Figure S1.** DTS Error Grid analysis of 2,616 post-calibration paired GLUCUBE–reference glucose measurements showing the distribution of values across clinical risk zones.
